# Granulated Bog Iron Ores as Sorbents in Passive (Bio)Remediation Systems for Arsenic Removal

**DOI:** 10.3389/fchem.2018.00054

**Published:** 2018-03-16

**Authors:** Klaudia Debiec, Grzegorz Rzepa, Tomasz Bajda, Witold Uhrynowski, Aleksandra Sklodowska, Jan Krzysztoforski, Lukasz Drewniak

**Affiliations:** ^1^Laboratory of Environmental Pollution Analysis, Faculty of Biology, University of Warsaw, Warsaw, Poland; ^2^Department of Mineralogy, Petrography and Geochemistry, Faculty of Geology, Geophysics and Environmental Protection, AGH University of Science and Technology, Krakow, Poland; ^3^Faculty of Chemical and Process Engineering, Warsaw University of Technology, Warsaw, Poland

**Keywords:** arsenic, mineral sorbents, bog iron ores (BIOs), dynamic sorption, water treatment, *in situ* remediation

## Abstract

The main element of PbRS (passive (bio)remediation systems) are sorbents, which act as natural filters retaining heavy metals and carriers of microorganisms involved in water treatment. Thus, the effectiveness of PbRS is determined by the quality of the (ad)sorbents, which should be stable under various environmental conditions, have a wide range of applications and be non-toxic to (micro)organisms used in these systems. Our previous studies showed that bog iron ores (BIOs) meet these requirements. However, further investigation of the physical and chemical parameters of BIOs under environmental conditions is required before their large-scale application in PbRS. The aim of this study was (i) to investigate the ability of granulated BIOs (gBIOs) to remove arsenic from various types of contaminated waters, and (ii) to estimate the application potential of gBIOs in technologies dedicated to water treatment. These studies were conducted on synthetic solutions of arsenic and environmental samples of arsenic contaminated water using a set of adsorption columns filled with gBIOs. The experiments performed in a static system revealed that gBIOs are appropriate arsenic and zinc adsorbent. Dynamic adsorption studies confirmed these results and showed, that the actual sorption efficiency of gBIOs depends on the adsorbate concentration and is directly proportional to them. Desorption analysis showed that As-loaded gBIOs are characterized by high chemical stability and they may be reused for the (ad)sorption of other elements, i.e., zinc. It was also shown that gBIOs may be used for remediation of both highly oxygenated waters and groundwater or settling ponds, where the oxygen level is low, as both forms of inorganic arsenic (arsenate and arsenite) were effectively removed. Arsenic concentration after treatment was <100 μg/L, which is below the limit for industrial water.

## Introduction

Passive (bio)remediation systems (PbRS) are low-cost and environmentally-friendly technological solutions for the treatment of metal-polluted water, which passes through a semi-permeable barrier while the contaminants are retained by means of biogeochemical reactions (Groudev et al., 2008; Obiri-Nyarko et al., 2014). Most of them are multi-reactive systems and involve: sorption, precipitation, as well as chemical and (micro)biological reactions (Macías et al., 2012; Ayora et al., 2016). Of these, sorption seems to be a key process as it is the basis of most of the passive remediation methods, affecting their efficiency (Kanel et al., 2006).

Adsorbents that are used in passive (bio)remediation systems (e.g., constructed wetlands or permeable reactive barriers) act as: (i) natural filters (barriers) retaining heavy metals by physico-chemical sorption, and (ii) carriers for microorganisms involved in water treatment (Li et al., 2013; Meng et al., 2014; Hua et al., 2015). Therefore, the effectiveness of PbRS is determined by the quality of the (ad)sorbents, which should be selected based on their range of applications and stability under various environmental conditions. Furthermore, there are two main physical and chemical requirements for adsorption materials: high surface area or volume of the micropores and fast kinetics of the adsorption reaction (Motsi et al., 2009). For example, an adsorbent with a high sorption capacity, but characterized by slow adsorption kinetics is not desirable due to long retention time and low efficiency. In turn, the use of an adsorbent with fast adsorption kinetics but with low sorption capacity is also inconvenient, as to ensure high efficiency, a large amount of the material is required. Finally, an adsorbent used in systems based on both physico-chemical and microbiological remediation technologies should also be non-toxic to bacteria.

One of the ways to reduce the impact of heavy metals on the biosphere is their permanent removal using sorbents, i.e., iron-based materials. Many laboratory studies have shown that iron-based sorbents (e.g., zero valent iron, ferrihydrite, schwertmannite, or magnetite) meet these requirements due to their high sorption capacity with regard to heavy metals and their short retention time (Rangsivek and Jekel, 2005; Violante et al., 2010; Dou et al., 2013; Maziarz and Matusik, 2017). Previous studies revealed that bog iron ores (BIOs) are a very promising sorbent of heavy metals with variegated surface chemistry and large specific surface area. High cation adsorption capacities were found, up to approximately 40 mg/g for Cu, 25 mg/g for Zn, 20 mg/g for Ni, 55 mg/g for Cr(III) and 97 mg/g for Pb, as well as anion adsorption capacities, up to 10 mg/g for chromate, 20 mg/g for arsenate and 35 mg/g for arsenite (Bajda et al., 2004; Bednarek et al., 2006; Rzepa et al., 2009; Tuchowska et al., 2016). There were also several successful attempts of using BIOs for *in situ* immobilization of heavy metals in soils and sediments, but only on a laboratory scale (Müller and Pluquet, 1998; Szrek et al., 2011). Furthermore, BIOs are characterized by high chemical stability and resistance to bioleaching (Debiec et al., 2017b). However, despite these properties, as well as their widespread availability and low operating cost, large-scale application of BIOs in passive remediation systems requires a detailed investigation of their usage under environmental conditions.

One of the most important issues is the analysis of the properties of sorbents using (artificial) solutions of heavy metals, the concentration of which reflects that in contaminated waters. Similar to other laboratory studies of iron-based sorbents, previous analysis of BIOs included static sorption analysis at relatively high concentrations of metals (e.g., up to 2700 mg/L of copper, 3,000 mg/L of lead, 3,000 mg/L of zinc and 360 mg/L of chromium; Rzepa et al., 2009), which are usually not found in the environment. Another aspect that should be considered is sorption capacity in the presence of various metal cations and other inorganic (e.g., sulfate, chloride or phosphate) (Chen et al., 2008; Chowdhury and Yanful, 2010) and organic compounds (e.g., citrate or aquatic humic acids) (Buschmann et al., 2006; Wang and Mulligan, 2006), as well as microbial biomass, which can influence the effectiveness of the process (Bauer and Blodau, 2006). Among the environmental factors which may also affect heavy metal sorption are physical parameters such as the temperature of contaminated water (Shipley et al., 2009) or pressure of water flowing through the adsorption system (Mohan and Pittman, 2007).

In this study, we aimed to answer the following questions: what is the actual potential of using BIOs as sorbents for arsenic removal and what are their actual properties when they applied in passive remediation systems on a commercial (industrial) scale. This study also presents the results of an investigation of the capabilities of arsenic-loaded adsorbent, including three-step chemical desorption experiments and studies of the re-use of As-loaded BIOs for the sorption of other heavy metals, in which zinc was used as an example. The properties of BIOs were analyzed using three types of natural arsenic-contaminated waters from various reservoirs located in the Zloty Stok area, SW Poland. All studies were performed with the use of the granulated bog iron ores (gBIOs), as they can be easily tested in a column flow system due to their higher permeability with regard to fine BIOs. The specific goals of the study include estimation of the: (i) chemical and physical parameters of gBIOs, (ii) efficiency of arsenic and zinc static sorption, (iii) sorption capacity with regard to arsenic by dynamic studies on environmental samples, (iv) stability of arsenic loaded sorbents, and (v) possibility for reusing gBIOs loaded with arsenic.

## Materials and methods

### Physical and chemical characterization of the contaminated waters

Water samples used in this study were collected in the Zloty Stok area (SW Poland), from the following reservoirs polluted with arsenic: (i) a settling pond of an old paint and varnish factory (settling pond–SP), (ii) a dewatering system of an ancient gold mine (surface water–SW), and (iii) a 30 m deep mine pit filled with water flowing through arsenic deposits (groundwater–GW). The total arsenic concentrations, as well as physical and chemical properties of investigated waters, are presented in Table 1.

**Table 1:** Physical and chemical characterization of groundwater (GW), surface water (SW) and settling pond (SP), including: concentration of cations and anions [μg/L], total organic carbon (TOC) [μg/L], pH and Eh [mV]).

	**GW**	**SW**	**SP**
Ag	<0.05	<0.05	<0.05
Al.	<0.50	1.10	1.10
As	313.00	2,341.00	5,282.00
As(III)	1.08	1.59	6.11
As(V)	311.92	2,339.41	5,275.89
B	<5.00	10.00	41.00
Ba	0.81	2.04	109.00
Be	<0.50	<0.50	<0.50
Cr	<2.00	<2.00	2.00
Cd	<0.05	0.06	<0.05
Cd	<0.05	0.06	<0.05
Cu	0.54	0.69	1.37
Fe	0.12	0.08	0.09
Li	1.60	6.50	7.00
Mn	<0.50	13.90	0.90
Mo	0.50	3.72	4.49
Ni	<0.50	0.90	0.70
Pb	<0.05	<0.05	0.36
Sb	0.24	4.30	4.60
Se	<2.00	<2.00	3.00
Sn	<0.50	<0.50	<0.50
Sr	25.40	86.30	122.00
Tl	<0.05	<0.05	<0.05
U	0.56	7.39	3.69
Zn	4.00	6.00	<1.00
V	<1.00	<1.00	2.00
Cl^−^	1,001.00	5,040.00	1,940.00
${\text{SO}}_{4}^{2-}$	83,000	96,000	70,000
${\text{NO}}_{3}^{-}$	1,825	2,141	1,294
${\text{PO}}_{4}^{3-}$	1,827	2,100	5,460
TOC	14,000	19,000	23,000
pH	7.18	7.48	7.62
Eh	170.20	170.90	165.30

The total concentrations of arsenic as well as other elements (Li, Be, B, Al, V, Cr, Mn, Co, Ni, Cu, Zn, Se, Sr, Mo, Ag, Cd, Sn, Sb, Ba, Tl, Pb, and U) in water were measured by inductively coupled plasma mass spectrometry (ICP-MS; ELAN DRC II, Perkin Elmer). Detection limit (DL) of this method for different elements is as a follows: 0.05 μg/L for Co, Mo, Ag, Fe, Sb, Tl, Pb, 0.5 μg/L for Al, Be, Mn, Ni, Cu, Sr, Sn, U, 1 μg/L for Li, V, Zn, Ba, 2 μg/L for Cr, Se, As, and 5 μg/L for B. Arsenic species were separated by ion chromatography on IonPac As18 (2 mm, Dionex) column on ICS Dionex 3000 instrument equipped with ASRS® 2 mm suppressor and coupled to a ZQ 2000 mass spectrometer (Waters). The eluent was introduced to the mass spectrometer (MS) via an electrospray source (DL 0.5 μg/L). Arsenic speciation was determined according to the method described by Debiec et al. (2017a). Chloride, sulfate, nitrate, phosphate and total organic carbon (TOC) concentrations were determined using Nanocolor® (Machery-Nagel GmbH, Germany) kits: DL 200, 10,000, 1,000, 200, and 2,000 μg/L, respectively. pH and Eh were measured using a multifunction meter (Elmetron CX-461) with compatible pH (Elmetron EPS1) and redox potential (Elmetron ER Pt-13) electrodes.

### Granulated bog iron ores

Granulated bog iron ores were obtained by mechanical granulation of the fine iron deposit with cement additives, consisting of a mixture of gypsum, REA-gypsum, anhydrite and/or granulated blast furnace slag. The size of BIOs granules ranged within 3–15 mm. Results of chemical composition of fine BIOs and gBIOs unloaded/loaded with arsenic are presented in Table 2. Moreover, unloaded as well as arsenic-loaded gBIOs were analyzed by X-ray diffractometry (XRD), Fourier-transform infrared spectroscopy (FTIR), scanning electron microscopy (SEM) and X-ray fluorescence (XRF) and subjected to thermal analysis. XRD patterns were collected using Rigaku SmartLab diffractometer equipped with a graphite monochromator and rotating Cu anode. FTIR spectra were collected using Thermo Scientific Nicolet 7600 spectrophotometer in the range 4,000–400 cm^−1^. Prior to analysis, KBr pellets were obtained by homogenizing 200 mg of ground KBr with 4 mg of the sample. Scanning electron microscope analyses were carried out in low vacuum mode, using an FEI 200 Quanta FEG microscope equipped with an EDS/EDAX spectrometer. The acceleration voltage was 15–20 kV. The samples were not coated with a conductive layer. Thermogravimetric (TG) and differential thermal analysis (DTA) measurements were performed using Netzsch STA 449F3 Jupiter apparatus. An air-dried sample was heated from 20° to 1,000°C, at the heating rate of 10°C/min^−1^ in flowing synthetic air. Analyses of the evolved gases were carried out using the quadrupole mass spectrometer Netzsch QMS 403C Aëolos.

**Table 2:** Chemical composition (wt. %) of natural and gBIOs, prior and after adsorption experiment.

	**BIOs**	**gBIOs**	**Loaded gBIOs**
SiO_2_	7.88	2.54	3.07
TiO_2_	0.02	0.69	0.70
Fe_2_O_3_	48.47	17.50	19.35
Al_2_O_3_	0.28	0.93	1.16
CaO	2.63	30.88	29.81
MgO	0.08	2.48	3.50
MnO	0.22	0.11	0.13
K_2_O	0.07	0.09	0.08
Na_2_O	0.04	0.04	0.05
P_2_O_5_	3.11	0.03	0.04
SO_3_	n.a.	17.87	14.35
As_2_O_3_	n.a.	b.d.l.	0.11
LOI	35.60	26.69	27.48

*LOI, loss on ignition; b.d.l., below detection limit; n.a., not analyzed*.

### Batch experiments

To estimate the maximum sorption capacity of gBIOs, batch sorption experiments were carried out. Due to the fact, that arsenic occurs mainly in the arsenate form in the contaminated water samples, batch sorption efficiency with respect to As(V), not As(III), was investigated. Furthermore, sorption efficiency of gBIOs with regard to zinc was also tested. These experiments allowed to determine the maximum sorption capacity of the adsorbent and to compare the sorption efficiency for both elements (Genç-Fuhrman et al., 2007).

Batch sorption experiments were performed using a series of separate solutions of arsenic (Na_2_HAsO_4_ × 7H_2_O) and zinc (ZnSO_4_) at the following concentrations: 5, 10, 20, 50, 150, 300, and 500 mg/L. Experiments were carried out in 500 mL flasks containing 400 mL of the metal solutions and 40 g of the sorbent. The suspensions were shaken (130 rpm) at room temperature for 2 h. According to our preliminary research (unpublished data), this time was sufficient for the adsorption reaction to reach equilibrium. After 2 h, 5 mL of the mixture was collected and centrifuged (5 min, 10,000 rpm), and then 4 mL of the supernatant was mixed with 69% HNO_3_ at a ratio of 4:1. Samples were stored at 4°C. Total arsenic and zinc concentrations were measured in the samples.

Based on the results of batch sorption experiments, adsorption efficiency was calculated. This parameter was calculated from the difference between the initial and final concentrations of the solutions, and the obtained values were converted to appropriate units (per kilogram of the adsorbent). Based on these results, Langmuir and Freundlich isotherms were calculated as described by Rzepa et al. (2009) according to the following formulas:

1. Langmuir isotherm: $\;\frac{C_{eq\;}}{S}=\;\frac{1}{qK}+\;\frac{C_{eq}}{q}$
2. Freundlich isotherm: $\log S=\log k+\;\frac{1}{n\;}\log C_{eq}$

Explanation of the abbreviations are given below.

*C*_*eq*_ – the equilibrium liquid phase concentration [mmol]

*S* – the amount of sorbent adsorbed per unit weight [mmol/kg]

*q* – sorption capacity [mmol/kg]

*K* – rate of adsorption [L/mmol]

*k* – constant related to the sorption capacity of the sorbent

$\frac{1}{n}$ – constant related to the sorption affinity of the sorbent.

### Dynamic sorption experiments

To further assess the sorption efficiency of the adsorbent, dynamic sorption experiments were performed. Experiments were carried out in three 1.5 L separate columns with an internal diameter of 57 mm. Each column contained 1 kg of the adsorbent, which was conditioned using tap water before the experiment to remove all loose binding fractions. Contaminated waters (GW, SW, SP) were fed to the columns from the bottom (against the force of gravity) using peristaltic pumps at a rate of 2.64 L/h, which corresponded to 20 min of retention time. Samples of water at the inflow and outflow of each column were collected once a day for 15 days and were stored at 4°C. This experiment was repeated twice.

**Figure 1:**
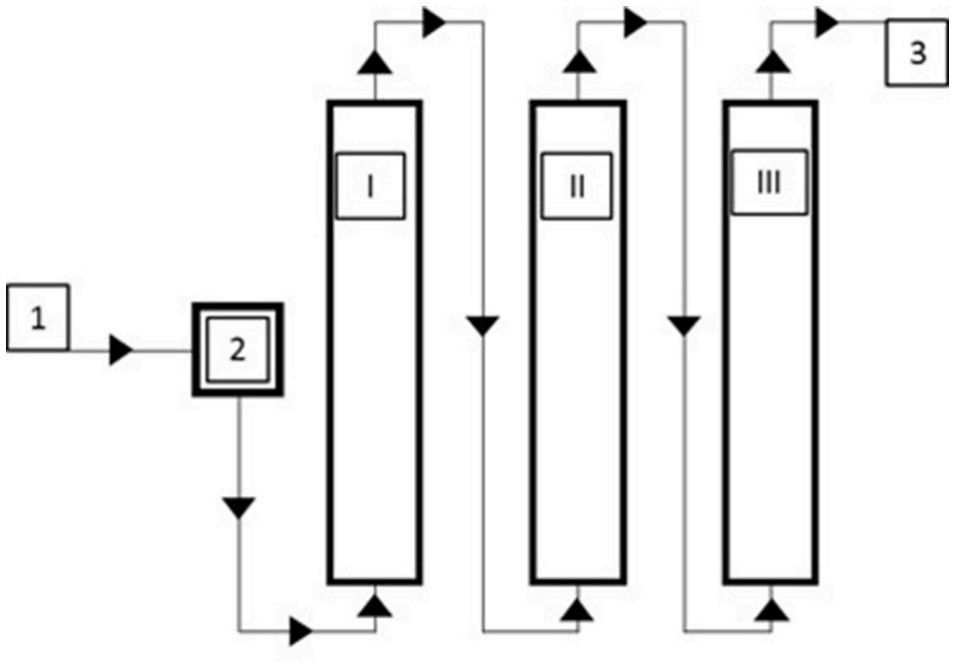
Scheme of the adsorption module for dynamic sorption studies 1/3–inflow/outflow, 2–pressure regulator and flowmeter, I–III–adsorption columns.

For surface water (SW), dynamic sorption experiments were carried out on a larger scale. The adsorption module consisted of a series of three 17 L columns filled with gBIOs (about 15 kg per column) (Figure 1). Samples of water were taken at four sampling points: before the adsorption module and after each column. Dynamic sorption experiments at various liquid flow rates (8, 20, and 40 L/h) were carried out. The slowest liquid flow through the adsorption module was also investigated in a long-term experiment (for 40 days). Total arsenic concentration was measured in the water samples.

### Desorption studies

The strength of arsenic bonding by gBIOs was tested in desorption experiments, which consisted of 3 subsequent steps. In the first step, 1 M MgCl_2_ (pH 8, reaction time of 2 h) was used. In the second and third steps, 1 M NaH_2_PO_4_ (pH 5) was used, with the reaction time of 16 and 24 h, respectively. The first step was repeated twice to check whether all the adsorbed As ions were removed from the deposit. The extractants used to remove the ion-exchanged species were selected according to the method by Keon et al. (2001). One gram portions of the materials were shaken in 50 mL of the appropriate solution for 2, 16, and 24 h, respectively. Between the subsequent washing steps, the materials were flushed 3 times with double-distilled water. After each washing step and centrifugation of the sample, the supernatant was analyzed for As.

### Re-use of gBIOs studies

To further assess the sorption potential of gBIOs, adsorption efficiency of the arsenic-loaded sorbent with regard to zinc was investigated. As-loaded granules samples were collected from dynamic sorption experiments carried out in 1.5 L columns (see section Dynamic Sorption Experiments). The sorbent was loaded with three types of arsenic contaminated water (GW, SW, SP). As-loaded sorbent samples were dried to constant weight and then used in subsequent batch experiments. Batch sorption experiments with arsenic-loaded gBIOs were performed in an analogous manner to those described in section Batch Experiments. In these studies, two zinc solutions were used: 10 and 300 mg/L. Total arsenic and zinc concentration were measured in the solutions samples.

### Chemical analysis

Mineralization of arsenic-loaded gBIOs after the sorption experiments was performed in 69% HNO_3_ and 30% H_2_O_2_, in a closed microwave system (Milestone Ethos Plus) at controlled thermal conditions (180°C) for 25 min. Total arsenic and zinc concentration in the samples were measured by atomic absorption spectrometry with an air-acetylene flame atomizer (TJA Solution, SOLAAR M, UK) (DL 1 μg/L). Arsenic and zinc standard solutions (Merck, Darmstadt, Germany) were prepared in 3% HNO_3_.

## Results and discussion

### Chemical characteristics of gBIOs

**Figure 2:**
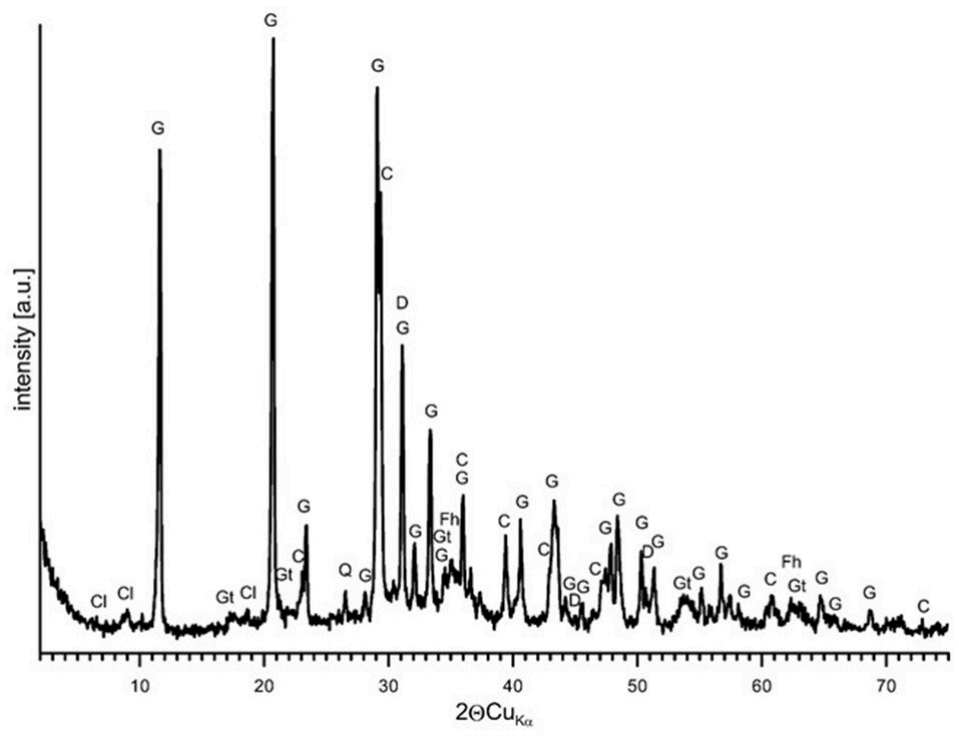
XRD pattern of the gBIOs. Explanations: G, gypsum; C, calcite; Gt, goethite; Fh, ferrihydrite; D, dolomite; C1, clay minerals.

**Figure 3:**
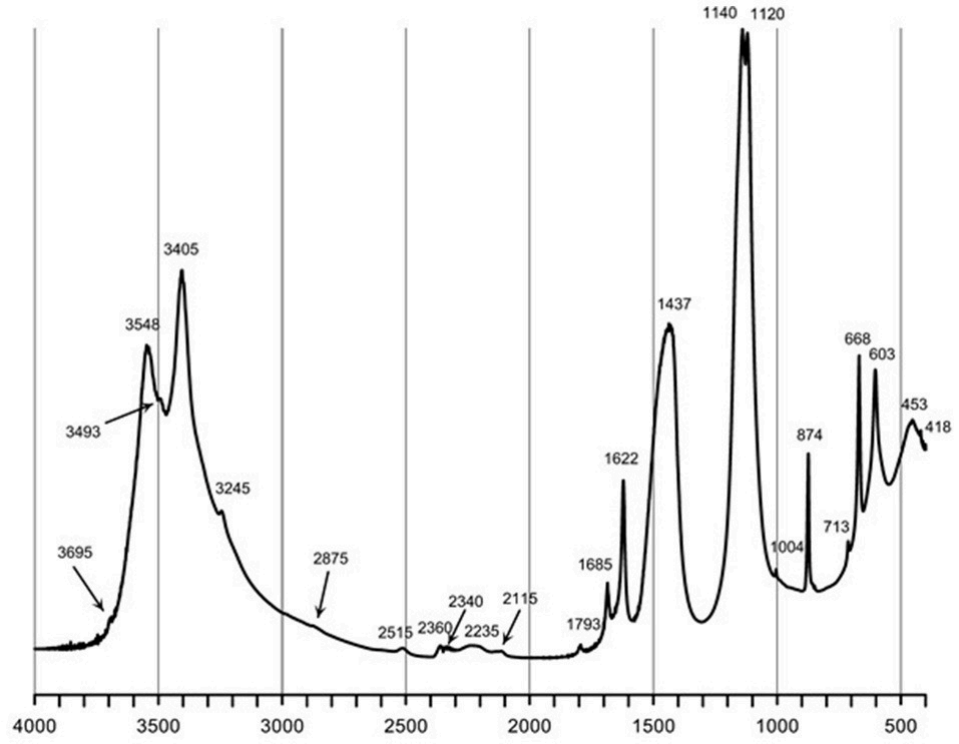
FTIR spectrum of the gBIOs. The numbers denote positions of the absorption bands.

**Figure 4:**
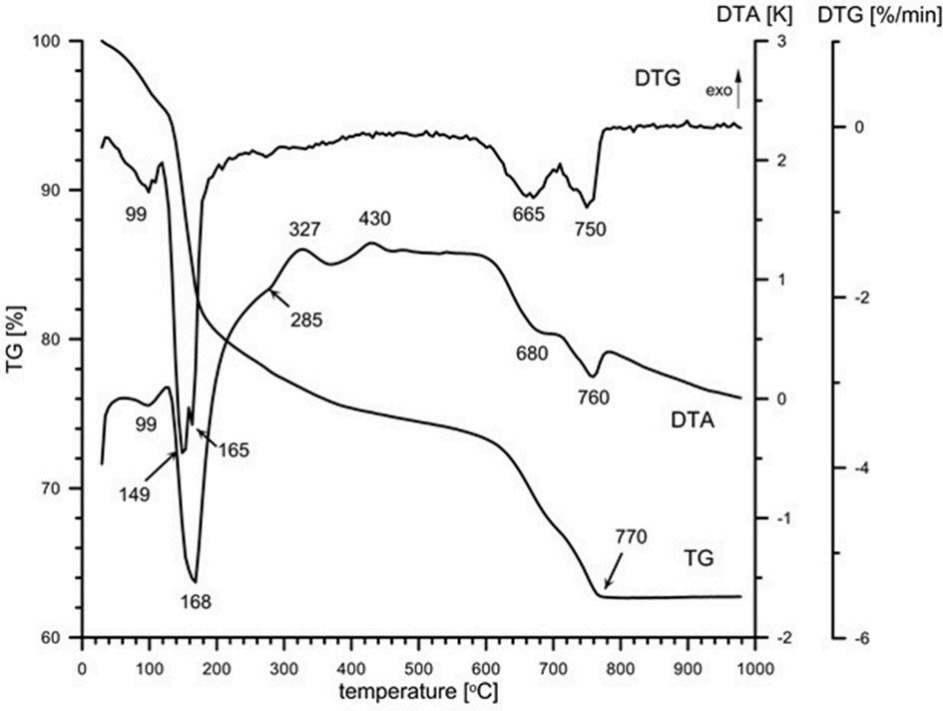
Thermal patterns (DTA–TG–DTG) and QMS signals (not to scale) of the gBIOs. The numbers denote temperatures of processes.

**Figure 5:**
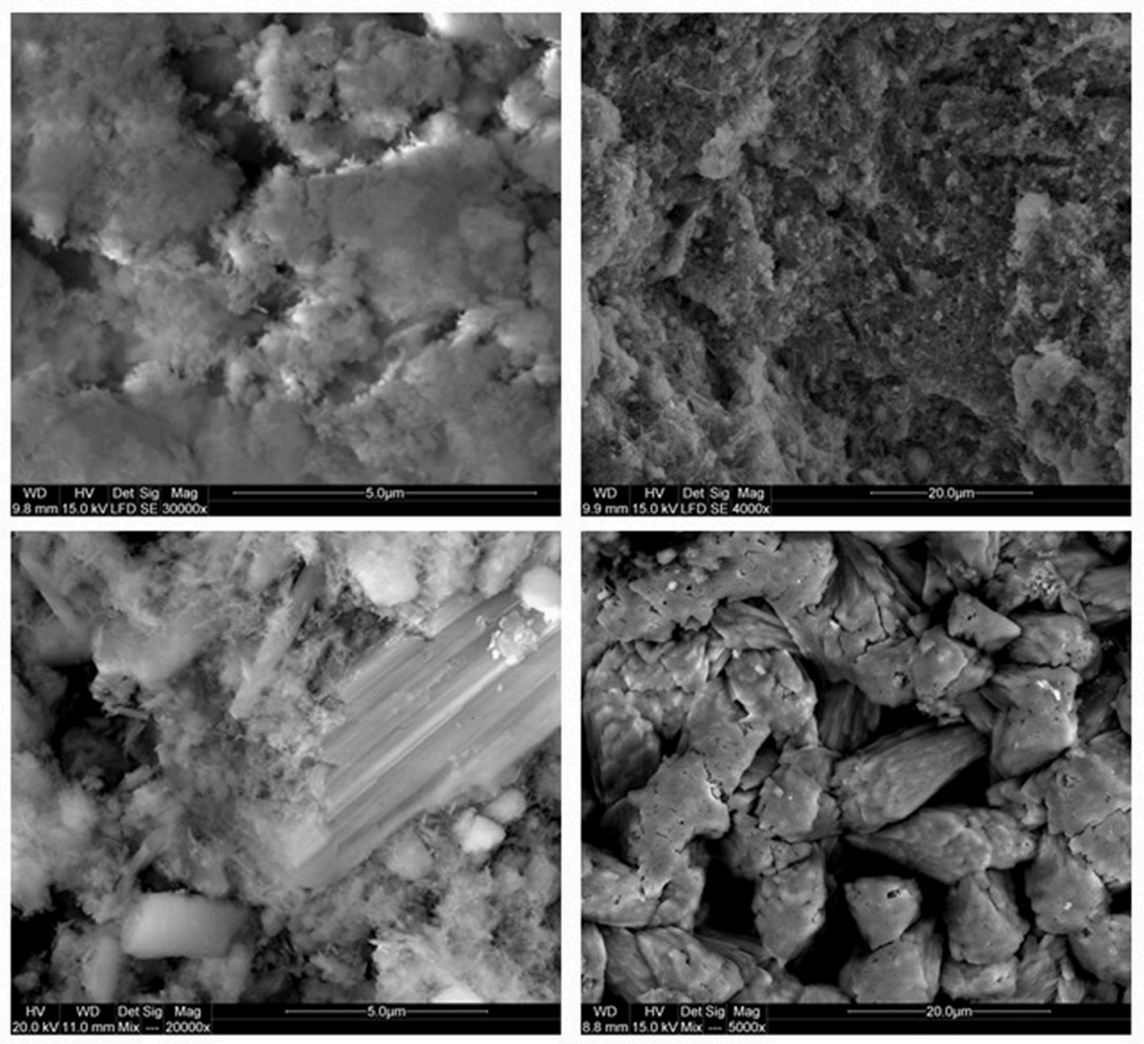
SEM photomicrographs of the gBIOs. Upper left: cryptocrystlline iron oxyhydroxide aggregates, upper right: minute carbonate crystallites embedded within ferruginous-organic mass, lower left: large gypsum and calcite crystals within microcrystalline iron oxyhydroxide, lower right: large calcite crystals on the surface.

Natural BIOs are composed mainly of iron oxyhydroxides: ferrihydrite and goethite as well as quartz and organic matter (Rzepa et al., 2009, 2016). The granulation process, however, seriously affects the ore composition–according to XRD and FTIR analyses (Figures 2, 3), gypsum and calcite predominate in the gBIOs, accompanied by quartz, iron oxyhydroxides and traces of clay minerals and dolomite. Thermal analyses showed the presence of organic matter admixture as well, which is oxidized in the temperature range of approximately 300–450°C (Figure 4). Among the inorganic components gypsum and carbonates dominate over iron oxyhydroxides. The content of the latter estimated based on the mass loss on dehydroxylation, is not more than 12 wt%. Scanning electron microscopic observations revealed that gBIOs is inhomogeneous (Figure 5). Usually, within cryptocrystalline ferruginous aggregates composed of ferrihydrite and goethite, numerous gypsum and calcite crystals are embedded. They might be either minute (up to several μm) or large (especially gypsum, up to 100 μm in size). In places CaSO_4_ and CaCO_3_ predominate and almost entirely mask the presence of the natural BIOs components. Mineralogical analyses are in a good accordance with chemical analyses. gBIOs is distinctly impoverished in iron compounds and phosphorus compared to the fine, natural BIOs. Silica and manganese contents are lower as well (Table 2). On the other hand, gBIOs contain over 10 times more CaO and approximately 30 times more MgO than the natural BIOs.

XRD patterns and FTIR spectra of gBIOs after adsorption experiments are virtually the same as those recorded for gBIOs prior to the experiments. Only EDS analyses suggest the presence of As on the surface of iron oxyhydroxides, but the arsenic content is barely higher than the limit of detection. This is supported by the results of XRF analyses which revealed the presence of As in the loaded gBIOs but not in the unloaded gBIOs (Table 2). These hardly noticeable changes resulted from the relatively low arsenic contents in the studied waters.

### Sorption properties of granulated bog iron ores

Mineral sorbents based on iron oxides and oxyhydroxides are characterized by various sorption capacities with regard to arsenic. Sorption efficiency may vary depending on the metal form (cation, oxyanion, etc.,) and its concentration. Cations are specifically adsorbed through interactions with a deprotonated surface hydroxyl group to form mono and binuclear inner-sphere complexes. Surface hydroxyl groups have also the capability of binding anions (like arsenate or chromate) via ligand exchange reaction, which displaces hydroxyl groups from coordination positions on the surface (Rzepa et al., 2009).

**Figure 6:**
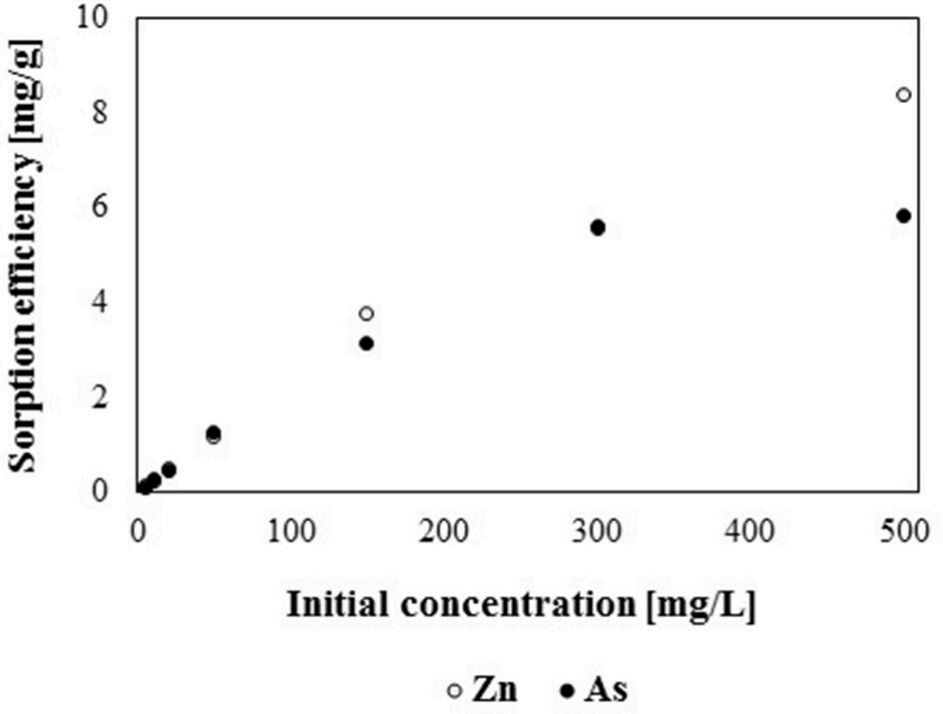
Sorption efficiency of gBIOs with regard to various initial concentration of As and Zn.

Batch sorption studies showed, that the sorption capacity of gBIOs depends on the type (anion or cation) and concentration of adsorbed metal (Figure 6). It was found, that the investigated sorbent is characterized by a higher sorption capacity with regard to zinc (cation) than arsenite (anion). The difference is especially prominent for solutions with metals concentration >100 mg/L. At lower arsenic and zinc concentrations, sorption efficiency for both elements was similar. These differences may be a result of lower cations exchange capacity (CEC) of mineral adsorbents than anions exchange capacity (AEC) values (Bajda, 2001). Sorption efficiency at low concentrations of arsenic and zinc is particularly desirable due to the fact that such amounts of metals may be found in contaminated waters in the environment. Although the investigation of sorption properties at high metal concentrations seems less significant from an environmental point of view, the obtained results may be used to compare the adsorption efficiency and maximum sorption capacity of various adsorbents with regard to the same compound.

As previously mentioned, the adsorption capacity of gBIOs is directly correlated with the initial concentration of the adsorbate. The higher initial adsorbate concentration the higher adsorption capacity. For the lowest studied concentration (5 mg/L), the adsorption capacity of gBIOs was 0.125 and 0.105 mg/g for zinc and arsenic, respectively, however in case of the highest studied concentration (500 mg/L) these values were 8.38 and 5.72 mg/g, respectively. Most of the available literature data concerned the research on determining the maximum adsorption capacity and studies of absorbent properties at lower heavy metals concentration are less common. Therefore, the maximum adsorption capacity of gBIOs with other adsorbent materials was compared. In case of zinc, literature data indicated many adsorbents which characterized by similar or inconsiderably higher adsorption capacity than gBIOs, i.e., carbon nanotubes−13.04–43.66 mg/g (for initial Zn concertation−1,000 mg/L) (Lu and Chiu, 2006), natural clay−11.02–75 mg/g (Zn 1,000 mg/L) (Veli and Alyüz, 2007), kaolin clay mineral−40,983 mg/g (Zn 50 mg/L) (Arias and Sen, 2009) or zeolites–about 13 mg/g (Zn 65 mg/L) (Perić et al., 2004). Various adsorption capacities of the adsorbents may be as a result not only adsorption properties of the particular adsorbents but also as various conditions of batch experiments, i.e., adsorbate initial concentration, retention time or volume of the adsorbent. It is also worth emphasizing that, the above comparison, concerns the granulated adsorbent (gBIOs) and raw materials (which were not granulated). The preparation of adsorbents to the application in flow conditions (granulation) requires the addition of various components to raw materials, which may effect on the decreasing of their adsorption capacity.

The As adsorption capacity of gBIOs was also compared with other materials. For example, adsorption capacity of zero valent iron (ZVI) amount to 0.221 mg/g at a low arsenic concentration (5 mg/L), being almost two times higher than that of gBIOs (Eljamal et al., 2013). However, at an arsenic concentration above 50 mg/L, gBIOs showed higher adsorption capacity than ZVI. Iron oxide minerals–magnetite, hematite, goethite, as well as Fe oxide-rich rock, laterite, revealed As adsorption capacities of 0.0495, 0.206, 0.0495, and 0.0495 mg/g, respectively for low (5 mg/L) arsenic concentrations (Aredes et al., 2012). Therefore, only hematite is characterized by higher adsorption capacity than gBIOs. Based on the above comparison (adsorption properties with regard to zinc and arsenic), it was shown that gBIOs exhibit relatively high adsorption capacity than other adsorbents and constitute an appropriate material to further research in flow systems.

To investigate the sorption isotherm, both Langmuir and Freundlich models were used. The *R*^2^ coefficient obtained for isotherms showed that the Freundlich model gives a better fit to experimental data for both arsenic and zinc (0.9581 and 0.9905, respectively) than the Langmuir model (0.8649 and 0.8915, respectively). Langmuir constants for arsenic were *q* = 108.7 and *K* = 7.08, however in case of zinc - 44.25 and 2260, respectively. Values of Freundlich constants for arsenic were: *n* = 1.95 and *K* = 53.02, while for zinc - 3.58 and 87.34, respectively.

### Dynamic sorption studies—verification of theoretical assumptions under environmental conditions

**Figure 7:**
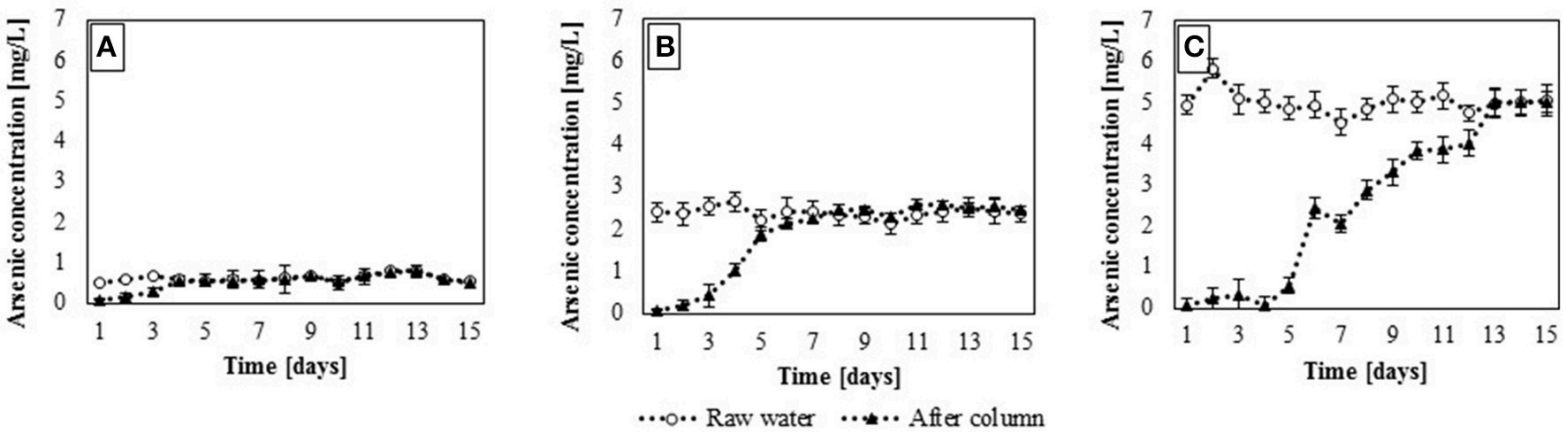
Dynamic sorption efficiency with regard to arsenic in the contaminated waters: **(A)**, groundwater; **(B)**, surfacewater and **(C)**, sediment pond (mean ± SD, *n* = 2).

The presence of heavy metals in contaminated water has a strong influence on the organisms living in such water as well as those exposed to its consumption (Fosso-Kankeu et al., 2011; Batvari et al., 2016). Concentrations of heavy metals in highly polluted waters are usually high from a biological point of view (strong impact on organisms) while their chemical content is actually low. As previously mentioned, adsorption efficiency depends on many factors, including the concentration of heavy metals in waters. Static sorption studies have shown that adsorption efficiency increases with the concentration of the adsorbate; thus, it seems its investigation is necessary, responding to the question what is the efficiency of the adsorbent in low heavy metals concentrations and at a constant flow of contaminated water. The dynamic sorption studies of gBIOs were carried out using samples of natural water contaminated with arsenic. These studies depicted the actual sorption capacity of the sorbent and reflected the actual interaction between adsorbent and adsorbate. The results are presented in Figure 7.

Dynamic sorption experiments confirmed the results obtained in batch sorption studies and showed that adsorption efficiency depends on the adsorbate concentration (Figure 7). In these experiments, the equilibrium saturation of the adsorbent was observed. Equilibrium saturation is regarded herein as the maximum saturation of the adsorbent at a given concentration of the adsorbate, resulting from the equilibrium between the adsorption and desorption processes. In groundwater, where arsenic concentration was the lowest (313 μg/L–average obtained form 15 days of experiments, *SD* = 1% ± 0.2, *n* = 15), equilibrium saturation of the adsorbent occurred after 4 days (Figure 7A). During that time, 253.44 L of contaminated water passed through the adsorption column. In the case of surface water and water from settling pond (containing 2,341 and 5,282 μg/L of arsenic (*SD* = 0.8% ± 0.2 and 1.3% ± 0.3, n = 15), respectively) sorption capacity of the adsorbent was higher and its equilibrium saturation occurred after 7 (443.52 L of contaminated water) and 13 days (823.68 L), respectively (Figures 7B,C). Thus, in the presence of higher initial arsenate concentrations in water, the equilibrium saturation occurred later than in the case of lower adsorbate concentration. This phenomenon may be a result of a limited sorption capacity of the adsorbent (Genç-Fuhrman et al., 2005).

In dynamic sorption study with the use of three types of arsenic contaminated waters, adsorption efficiency of gBIOs was also calculated. It was shown that in groundwater, adsorption efficiency of gBIOs was the lowest (0.0013 mg/g). In the cases of surface water and water from settling pond, adsorption efficiencies were 0.0085 and 0.0364 mg/g, respectively. These results confirmed the relationship observed in batch sorption experiments, that adsorption efficiency depends directly on the adsorbate concentration. In flow system experiments, was also shown, that adsorption efficiency of the adsorbent increased with the initial arsenate concentration. Moreover, adsorption efficiencies calculated for the dynamic system are actually lower than those estimated in batch experiments. In the case of the solution with arsenic concentration 5 mg/L (batch experiments), gBIOs adsorption efficiency was 0.105 mg/g, and it was almost three times higher than in the case of water from the settling pond with a similar arsenic concentration. This phenomenon may be explained by too short retention time to reach equilibrium saturation or the physical/chemical instability of the sorbent under constant flow conditions (Maji et al., 2008; Ali, 2012; Ge et al., 2014; Matouq et al., 2015).

Despite the fact that adsorption capacities of adsorbents are lower in dynamic conditions than in batch experiments, the studies in flow systems are particularly important from their application point of view. There are numerous solutions dedicated to arsenic removal from contaminated water based on the flow single-column systems. According to Guo et al. (2008), an appropriate arsenic adsorbent was activated siderite-hematite filter, which purified water for 20 days (flow rate 0.5 mL/min, initial arsenic concentration−0.5 mg/L). Other examples were hydrous ferric oxide incorporated diatomite, which allowed reducing arsenic concentration from 341 to 1.0 mg/L (Jang et al., 2007) or iron filings which enabled to reduce the initial arsenic concentration of 1.5 mg/L up to 85.0% (Takanashi et al., 2004). Although above mentioned adsorbents revealed high adsorption efficiency, they were tested only in laboratory scale, with the use of significantly smaller columns than in our study. Thus, it is difficult to directly compare properties of gBIOs with literature data. However, considering the reduction of arsenic concentration in all type of contaminated water, percentage adsorption efficiencies were calculated. These efficiencies appeared to depend on initial arsenic concentration in water. For groundwater, surface water and water from settling pond, arsenic concentration reductions were 82, 96, and 98%, respectively. Therefore, one can conclude, that despite significantly lower adsorption efficiency of gBIOs in flow system than in batch experiments, the efficiency is still high in comparison to other adsorbents studied in dynamic systems.

Differences in the adsorption efficiency in various types of contaminated water can also depend on their physical and chemical properties, including arsenic speciation (Morillo et al., 2015), which in turn depends mainly on the pH and redox conditions (Shipley et al., 2009; Mamindy-Pajany et al., 2011). At an environmentally relevant pH 5–7, arsenite exists as an undissociated species (HAsO_2_) while arsenate exists as an oxyanion (H_2_${\text{AsO}}_{4}^{-}$, ${\text{HAsO}}_{4}^{2-}$). For this reason, the oxidized form is more readily removed from contaminated waters by common techniques (i.e., adsorption, ion exchange, etc.) due to its charge (Mohan and Pittman, 2007). Literature data showed that iron-based mineral sorbents are characterized by high arsenic sorption efficiency in the pH range of 3.5–9.5 (Zhu et al., 2009). Below pH 3, sorption efficiency with regard to both arsenic forms is significantly decreased due to the increased solubility of iron oxyhydroxides (Zeng, 2004). In the case of the investigated types of waters, pH values are in the range of 7.18–7.62 (Table 1). Therefore, arsenic occurs mainly in arsenate form and, under these conditions, adsorption efficiency is high.

Ion competition for adsorption sites can also affect the efficiency of sorption of arsenic and zinc. Moreover, the presence of other heavy metals, as well as organic compounds in the contaminated water, can reduce sorption ability of iron-based sorbents, as they may occupy the binding sites and block them for arsenic or zinc species. In the case of the investigated samples, the effect of heavy metals is negligible, as the heavy metals concentrations (such as Zn, Mn, Cr, Ni, Cu, Cd, Pb) in GW, SW, and SP are low (Table 1). The small amounts of organic compounds (14.0, 19.0, and 23.0 mg/L of total organic carbon for GW, SW, and SP, respectively) (Table 1) also have no significant influence on the sorption capacity and efficiency of the adsorbent (Genç-Fuhrman et al., 2016).

On the other hand, elevated concentrations of anions can also affect the adsorption efficiency, and in the case of arsenic adsorption, the effect of co-occurrence of phosphates may be especially significant (Zeng et al., 2008). This results from the similarity of arsenate and phosphate, which are both tetrahedral oxyanions that can be specifically adsorbed on Al and Fe oxides (Liu et al., 2001). In the case of zinc adsorption, elevated concentration of sulfate can decrease adsorption efficiency, due to the formation of metal–anion complexes with low solubility (Avery and Tobin, 1993). The presence of sulfate may also influence arsenate adsorption, but their bonding strength with iron (hydr)oxides is much weaker than for arsenic (Zhu et al., 2009). The concentrations of phosphate and sulfate in the investigated waters are significantly higher than arsenic concentration (Table 1). Thus, their presence may reduce arsenic adsorption efficiency, but this relationship was not investigated in this study.

### Application of the adsorption column module in pilot scale experiments

Dynamic sorption studies carried out in columns showed that such adsorbers may be successfully used to assess sorption parameters of the pellets. Experiments performed in small columns allowed to (i) estimate the actual sorption capacity of the sorbent at low arsenic concentration (with the use of natural As-contaminated water), (ii) investigate the sorption efficiency in dynamic sorption systems and (iii) determine the retention time of the contaminated water, required for optimization of the process. These results contributed to the development of columns for the pilot-scale adsorption module, which was used to increase the scale of treatment of arsenic-contaminated water. This module consists of a series of three large columns filled with the iron sorbent, which was tested for the selected type of contaminated waters (surface water, containing approximately 2.5 mg/L of arsenic). Adsorption module was used to conduct short- as well as long-term experiments. In the short-term experiments, various liquid flows were tested. The results are presented in Table 3.

**Table 3:** Total arsenic concentration [mg/L] in untreated water (raw) and after each column of the adsorption module at various liquid flows.

**Liquid flow] [L/h]**	**Raw water**	**After 1st column**	**After 2nd column**	**After 3rd column**
40	2.666	0.108	0.010	<LQM
20	2.61	0.076	0.004	<LQM
8	2.849	0.036	<LQM^*^	<LQM

* *LQM - result below the limit of detection by analytical method used (< 0.001 mg/L)*.

It was found, that the adsorption columns module efficiently purified water contaminated with arsenic. In short-term experiments, the final arsenic concentration (after the third column) was below 0.01 mg/L (Table 3). The efficiency of arsenic removal depended on the flow rate, and directly correlated with the retention time in the columns. In the variant with the highest water flow (1 m^3^/day = 40 L/h), the retention time was the shortest; thus, the concentration of arsenic in water decreased more slowly than in the experiments with lower flow rates and longer retention times. The highest efficiency of the adsorption module was noted for the slowest flow rate of water (0.2 m^3^/day = 8 L/h).

**Figure 8:**
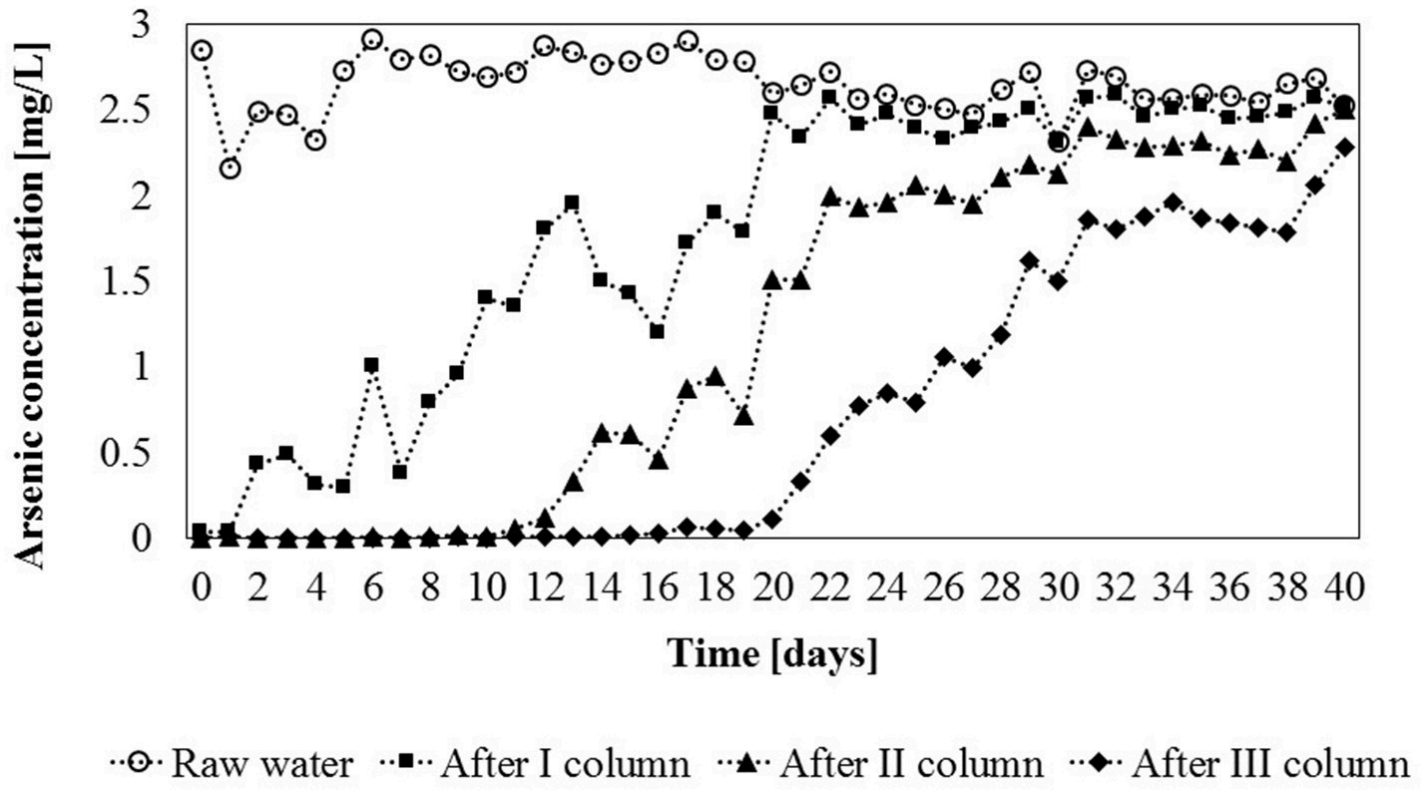
Total arsenic concertration [mg/L] in untreated water and after each column of the installation in constant flow experiments (flow rate = 8 L/h) (measurement deviation 0.6%).

Adsorbent particles are tightly packed in the column and form a bed of a certain height. Contaminated water can be introduced into the column filled with sorbent granules, on which adsorption process occurs. During an adsorption cycle, the zone with a higher metal concentration is first formed in the introductory part of the column. Therefore, the mass transfer between the liquid and the adsorbent occurs only in a part of the column. This part is named: the mass transfer zone (MTZ). If metal concentration in water is permanently measured at the outflow of the column, the transfer of the compounds adsorbed on the bed is observed only when the MTZ is close to the end of the adsorbent bed. Based on the analysis of the changes in the concentration of the adsorbate at the outflow of the column, in time it is possible to present the breakthrough curve for the adsorbent (Thomas and Crittenden, 1998). Such analysis was carried out for the pilot-scale adsorption module and the obtained breakthrough curves for each column (Figure 8).

It was found, that the first column was saturated (equilibrium saturation) after 20 days of the experiment. During this time, 4 m^3^ of water passed through the column. The adsorption capacity of gBIOs in this column was the highest and was 2.58 mg/kg. The second column was saturated within the next 11 days, after the total of 6.2 m^3^ of water had passed through the column (adsorption capacity was 1.65 mg/kg). The last column in the module was saturated after next 9 days (total of 8 m^3^ of water). Thus, equilibrium saturation of the sorbent in the entire module was observed after 40 days of the experiment. In the third column, the concentration of As in the inflowing water was the lowest, thus the lowest adsorption capacity was observed (1.44 mg/kg). This means that the actual adsorption of gBIOs in the entire module was approximately 1.89 mg of as per kilogram of the adsorbent.

The actual sorption efficiency in the dynamic system was significantly lower than that indicated by the results of batch sorption experiments. These showed that the theoretical gBIOs sorption capacity for 2.5 mg/L of arsenic (initial concentration) was 62.22 mg per kilogram of the adsorbent. This value is more than 30-fold higher than that obtained in the dynamic sorption study. Therefore, adsorption efficiency depends not only on adsorbate concentration but also on the type of adsorption process (static/dynamic processes) and adsorption reaction time (retention time in the adsorber).

Nevertheless, it was shown that, during the first 20 days of the experiment (long-term experiment), the total arsenic concentration in water samples (after the third column) was less than 100 μg/L, which is the upper limit for industrial water (according to the Regulation of Polish Ministry of the Environment, 2006). Thus, the adsorption module can be used for the purification of technological water according to the required concentration.

Many available technologies for arsenic removal from contaminated water were reported in the literature, but none of them gained a dominant position (EPA, 2014). Most of these technologies require a permanent adaptation to the local environmental conditions and to increase their efficiency, it is necessary to their redesign or connection with others available methods. Adsorption technology based on gBIOs has a high application potential since the method requires only adjustment to the application scale while design changes or complement by other methods are redundant. The economic and financial analysis showed that capital and operation costs (per year, including adsorbent costs) for the installation of 60 m^3^/h were attributed to relatively low (Kowalczyk, 2015). In the United State Environmental Protection Agency report (EPA, 2014) capital and operation costs of some remediation methods (i.e., reverse osmosis, coagulation, ion exchange as well as ZVI and ferrihydrite adsorption) were described. For example, the capital cost for reverse osmosis (water flow rate 13.5 m^3^/h) was estimated at almost 100 times higher than gBIOs-based technology, however, the operation costs were more than two times lower. For coagulation (2,000 m^3^/h), capital cost was two times higher than the gBIOs-based system, while the operation cost was three times lower. In case of ion exchange (158 m^3^/h) it was estimated to require low capital costs but operation costs were more than three higher than the proposed technology. On the other hand, gBIOs-based adsorption system was very cost-effective in comparison with other adsorption-based methods. For example, application of ZVI, as well as adsorption of ferrihydrite (158 m^3^/h), requires significantly higher capital cost than gBIOs-based system (almost two orders of magnitude) and more than two times more of operation cost. Therefore, application of the gBIOs adsorption system seems to be justified not only due to the high water purification efficiency but also for the economic reasons.

### Chemical stability of the adsorbent

To estimate the chemical stability of arsenic-loaded BIOs, three-step desorption experiment was performed. The strength of arsenic binding on the gBIOs surface has a particular significance in the context of their large-scale application in the treatment of arsenic contaminated water. High chemical stability of the adsorbent is required from the point of view of potential storage of As-loaded gBIOs as a toxic waste.

**Figure 9:**
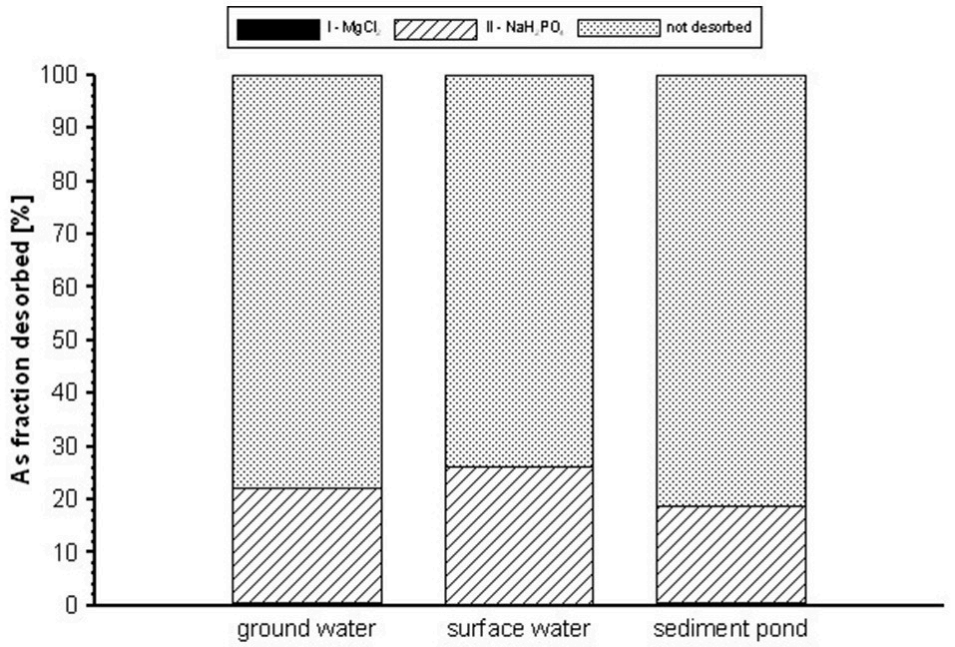
Desorption of As from the gBIOs.

The results of the II and III steps of desorption experiment were presented as the sum of As concentrations in the eluent after the extractions. Regardless of the type of contaminated waters, complete arsenic desorption was not achieved (Figure 9). In the first washing step, the desorption was insignificant, since only 0.09–0.22% of the total arsenic was removed after 2 h of the reaction with MgCl_2_. Washing with NaH_2_PO_4_ (II step) removed a further 9.7, 14.9, and 3.5% of the adsorbed As from the gBIOs reacted with GW, SW, SP, respectively. Based on the results, it was estimated that 85–96% of the adsorbed arsenic is permanently bound to the material. Permanently bound arsenic had apparently co-precipitated with amorphous and crystalline Fe-oxyhydroxides found in the gBIOs. Therefore, this sorbent is characterized by high chemical stability with regard to adsorbed compounds, especially those bound by crystalline Fe oxyhydroxides. Amorphous or poorly crystalline oxyhydroxides (i.e., ferrihydrite) are less stable, but the surface is stabilized by the presence of adsorbed species (Cornell and Schwertmann, 2003), hindering they possible transformation.

### Re-use of bog iron ore granules

**Figure 10:**
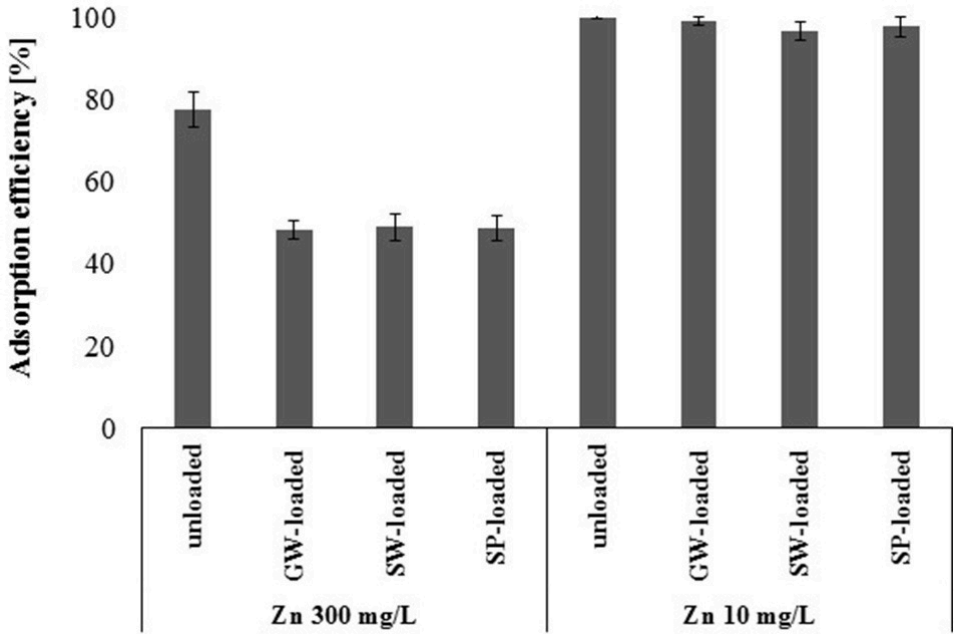
Adsorption efficiency of the unloaded and ground water-(GW), surface water-(SW), and sediment pond water (SP)-loaded gBIOs with regard to zinc at two concentrations (mean ± SD, *n* = 3).

Batch sorption experiments showed that the sorption capacity of gBIOs was significantly higher than indicated by the dynamic sorption results. Arsenic-loaded gBIOs after dynamic sorption studies have many unoccupied binding sites, which, due to the thermodynamic equilibrium, cannot be used in arsenic adsorption processes (in waters with similar or lower arsenic concentration). The question therefore arises, whether the unoccupied binding sites can be used for the adsorption of other heavy metals. In this study, experiments were performed on the example of arsenic-loaded gBIOs and two selected zinc solutions with the concentrations of 10 and 300 mg/L (Figure 10). Batch sorption experiments on unloaded gBIOs revealed that complete saturation was achieved when 300 mg/L of zinc concentration was used. However, when zinc concentration was 10 mg/L, we observed the highest adsorption efficiency, what suggests, that sorbent has not been fully saturated. Thus, these zinc concentrations were selected for the re-use of gBIOs experiments.

The experiments involving the re-use of the adsorbent showed that arsenic-loaded gBIOs are capable of adsorption of zinc at both concentrations. Adsorption efficiency of the unloaded sorbent was higher than the arsenic-loaded deposit. Nevertheless, this difference was significant only in the case of the higher zinc concentration. The decrease in the efficiency of adsorption in the presence of higher zinc concentration (300 mg/L) was 18%, while in the presence of the lower zinc concentration (10 mg/L) it was approximately 2%. Moreover, the adsorption efficiency of the arsenic-loaded sorbent with regard to zinc was similar regardless of arsenic content in the sorbent.

To assess the possible desorption of arsenic from the ore caused by the zinc solutions, total arsenic concentration was measured in arsenic-loaded gBIOs before and after the experiment. Before the experiment, the sorbent saturated with the groundwater (GW) contained 50.6 mg/kg ± 4.2 of arsenic, surface water (SW)−178.1 mg/kg ± 6.0, and water from settling pond (SP)−518.9 mg/kg ± 23.8. It was found that release of arsenic from iron-based adsorbent throughout the experiment was inconsiderable and the concentration of this element in the solution did not exceed 6% of content arsenic adsorbed by gBIOs, which corresponds to a maximum concentration of arsenic of 0.0958 mg/L. This arsenic concentration is below the upper limit of arsenic concentration for industrial waters. Low desorption degree indicates that arsenic is strongly bound to the surface of the sorbent, and that the sorbent is characterized by chemical stability, what was confirmed by the results of three-steps chemical desorption results described in section Chemical Stability of the Adsorbent. Chemical stability of As-loaded gBIOs have a particular significance from an environmental point of view, as desorption of the adsorbed metal from sorbents is particularly disadvantageous due to the risk of (re)contamination of the purified water.

## Conclusions

Despite the fact, that modification of bog iron ores (granulation) caused significant changes in the mineralogical structure and adsorption properties, it was shown that granular BIOs may be used as an arsenic adsorbent. Furthermore, chemical characterization of gBIOs showed an insignificant difference between the unloaded and As-loaded adsorbent. Therefore, saturation of gBIOs with arsenic has no significant influence on gBIOs properties.

The experiments performed in both static and dynamic systems revealed that gBIOs are an appropriate arsenic and zinc sorbent. This iron-based deposit is characterized by a high sorption capacity as well as short retention time with regard to both elements. However, arsenic concentrations in the contaminated water have a significant effect on the adsorption efficiency of the sorbent. At low arsenic concentration (<10 mg/L), adsorption efficiency is significantly lower than at higher concentrations.

Batch sorption experiments allowed to determine the maximum sorption capacity of the adsorbent and to compare the efficiency of adsorption of various elements. However, to investigate the actual adsorption efficiency in the presence of low arsenic concentrations, as well as the actual applicability of BIOs for the treatment of natural waters contaminated with arsenic, dynamic studies were necessary.

The performed experiments indicated that arsenic-loaded gBIOs may be reused for the adsorption of other elements i.e., zinc so that it is possible to increase the applicability of the adsorbent. Desorption studies showed that arsenic-loaded sorbent is characterized by high chemical stability; thus, there is no risk of release of the adsorbed arsenic and re-contamination of water. Based on the obtained results, it was shown that the iron-based sorbent may be successfully used in adsorption-based (bio)technologies dedicated to the treatment of arsenic contaminated water, including passive (bio)remediation systems.

The maximum utilization of the sorption potential of an adsorbent is important for both ecological and economic reasons. Complete saturation of the adsorbent (without leaving any unoccupied binding sites) may contribute to the reduction in the consumption of the sorbent and lowering of the cost of treatment of waters contaminated with heavy metals.

## Author contributions

KD was involved in planning and executing most of the experiments and wrote the manuscript; GR and TB were involved in the investigation of gBIOs properties, desorption studies, data development and article preparation; WU participated in dynamic sorption experiments and was involved in article preparation; AS was involved in article preparation; JK was involved in consultation of article details; LD was the head of the project and was involved in consultation and article preparation. All authors read and approved the final manuscript.

### Conflict of interest statement

The authors declare that the research was conducted in the absence of any commercial or financial relationships that could be construed as a potential conflict of interest.
